# Severe secondary hyperparathyroidism in patients on haemodialysis is associated with a high initial serum parathyroid hormone and beta-CrossLaps level: Results from an incident cohort

**DOI:** 10.1371/journal.pone.0199140

**Published:** 2018-06-18

**Authors:** Guillaume Jean, Marie Hélène Lafage-Proust, Jean Claude Souberbielle, Sylvain Lechevallier, Patrik Deleaval, Christie Lorriaux, Jean Marc Hurot, Brice Mayor, Manolie Mehdi, Charles Chazot

**Affiliations:** 1 NEPHROCARE Tassin-Charcot, Haemodialysis, Sainte Foy-les-Lyon,France; 2 Institut National de la Sante et de la Recherche Medical (INSERM) 890, CHU, Saint-Etienne, France; 3 Université Paris Descartes, Inserm U845, and Hôpital Necker, Service d’explorations fonctionnelles, Paris,France; 4 CERBALLIANCE, Laboratoire de biologie médicale du Grand Vallon, Sainte Foy-les-Lyon, France; 5 F CRIN INI-CRCT (Cardiovascular and Renal Clinical Trialists), Nancy, France; The University of Tokyo, JAPAN

## Abstract

**Background:**

Secondary hyperparathyroidism (SHPT) is a frequent complication of renal disease and most commonly occurs in patients on haemodialysis (HD) with metabolic, vascular, endocrine, and bone complications. The aim of this study was to analyze the evolution of mineral metabolism parameters during the first 36 months of HD treatment and identify the initial factors associated with severe SHPT.

**Methods:**

Serum parathyroid hormone (PTH), calcium and phosphate levels were measured monthly; bone-specific alkaline phosphatase (b-ALP) and beta-CrossLaps (CTX) were measured biannually. Severe SHPT was defined as the need for cinacalcet treatment. Patients with less than 24 months of follow-up were excluded.

**Results:**

One hundred thirty-three incident HD patients were included. Baseline mean PTH was 275 ± 210 pg/mL. After an initial drop at the third month (172 ± 133 pg/mL), the serum PTH level progressively increased to the maximum at 36 months (367 ± 254 pg/mL). This initial drop was associated with the initial correction of both hypocalcaemia and hyperphosphataemia. Serum CTX and b-ALP revealed no significant changes over time. Severe SHPT was observed in 18% of patients and was associated with higher mean calcaemia and phosphataemia. In logistic regression, the initial factors associated with the risk of severe SHPT were: female sex, higher baseline PTH and CTX values. A receiver operation characteristic curve analysis identified a cut-off value of >374 pg/mL for baseline PTH and >1.2 μg/L for CTX for increased risk of developing severe SHPT. The relative risk of developing severe SHPT was 3.7 (1.8–7.5, p = 0.002) for high baseline CTX, 4.9 (2.4–9.7, p = 0.001) for high baseline PTH, and 7.7 (3.6–16, p< 0.0001) when both criteria were present.

**Conclusion:**

After an initial drop, a progressive increase in the serum PTH level during the first 3 years of HD treatment was observed despite aggressive therapy. High baseline levels of PTH and CTX increased the risk of developing severe SHPT.

## Introduction

In patients with chronic kidney disease (CKD) and more specifically those on haemodialysis therapy (HD), secondary hyperparathyroidism (SHPT) in frequently observed as reported in French and European dialysis cohorts [1,2]. Together with effects of SHPT on bone, such as an increase in the bone turnover rate and increased risk of fractures [3], the main consequences of SHPT are cardiovascular calcification [4] and a higher mortality rate [5].

It has been recognized that SHPT worsens when the estimated glomerular filtration rate (EGFR) decreases [6]. In order to prevent this change in CKD stages 3 to 5, the Kidney Disease Improving Global Outcomes (KDIGO) experts recommended that the serum parathyroid hormone (PTH) level should be maintained in the normal range of the assay [7], even if the optimal level is not known.

In CKD stage 5D, in order to account for the unpredictable effects of PTH on bone, maintaining a mild state of SHPT has been recommended to avoid a low bone turnover rate, which is a risk factor for adynamic bone disease (ABD). However, the serum PTH level is not a good marker of the bone turnover rate. From bone histology studies, we only know that severe SHPT can be excluded when serum PTH levels are within the normal range, and that ABD can be excluded when the serum PTH level is 9 times above the upper limit of the normal range [8]. Between these two limits, normal bone, SHPT and ABD can be observed. Measurement of serum bone markers, such as alkaline phosphatases, could be useful to provide a more robust measure of bone status.

In addition, a serum PTH cut-off level as high as 9 times the upper limit of the assay may lead to undertreatment of patients with SHPT having a PTH level below this limit. At this point, owing to the progressive resistance of SHPT to standard therapies [9], nephrologists need to use calcimimetics or parathyroidectomy (PTX) to treat more severe SHPT. This strategy is expensive and potentially harmful.

Little is known about the changes of the initial serum PTH level in patients who are new to HD therapy, and it is unclear if the initial PTH value, which is a surrogate of CKD care before dialysis, affects the mid-term risk for developing severe SHPT. The same is true for serum bone markers such as bone-specific alkaline phosphatases (b-ALP) and beta-CrossLaps (CTX).Thus, we performed an observational study in incident HD patients in order to address these questions, as well as analyse changes in serum calcium, phosphates, PTH, b-ALP and CTX.

## Methods

All patients having their first HD session at our institution between January 2009 and January 2013 were included if they were followed by one of the co-authors of NephroCare Tassin-Charcot for more than 3 months. Exclusion criteria were a previous PTX, cinacalcet, or antiosteoporotic therapy and a follow-up period of less than 2 year after dialysis onset.

Patients meeting our eligibility criteria were enrolled in the study and observed until January 2016. The study was conducted in compliance with the ethical principles of the Declaration of Helsinki and was approved by our local Nephrocare ethic committee. All patients provided their signed consent to have their data entered in a database for analysis.

The following patient information was recorded: medical history, including cardiovascular events and risk factors; pharmaceutical treatment, including vitamin D, cinacalcet, and phosphate binders; and baseline results from standard serum laboratory tests just before the first dialysis session. Serum levels were sampled monthly to measure the following: PTH, which was determined using a second-generation assay (ElecSysG; Roche Diagnostics, Meylan, France, reference value 10–65 pg/mL), calcium, phosphorus, albumin, and C-reactive protein (CRP). Every 6 months, we also measured serum β-CrossLaps (CTX, chemiluminescent assay, Roche, Basel, Switzerland, reference value < 0.7 μg/L), bone-specific alkaline phosphatase (b-ALP, chemiluminescent assay, Beckmann Inc., Urbana, USA, reference value 3–20.9 μg/L) and 25-hydroxyvitamin D (25-OHD, Architect, Abbott Laboratories. Abbott Park, IL, USA.). For laboratory tests, blood samples were obtained in a non-fasting state before a mid-week dialysis session, and all serum levels were measured from the same blood draw. Common laboratory analyses were performed by the Grand Vallon Laboratory (NOVESCIA Lyon, France).

Severe SHPT was defined as the need for cinacalcet treatment. The criteria for cinacalcet prescription were as follows: persistent serum PTH level above 800 pg/ml for more than 6 months despite implementation of maximum vitamin D and calcium therapies, or both serum PTH above 400 pg/ml and persistent hypercalcaemia, or PTH above 400 pg/ml and increased serum bone markers (b-ALP > 25 μg/L or CTX > 4 μg/L).

Patients received dialysis three times weekly, 4 to 8 hours per session, using polysulfone high-flux filters (FX 60, 80, 100, 800 and 1000; Fresenius Medical Care, Bad Homburg, Germany) in HD or post-dilution online haemodiafiltration (HDF). The blood flow rate ranged between 220 and 400 ml/min, and the dialysate flow rate ranged between 350 and 800 ml/min. The standard dialysis calcium concentration (DCC) was 1.5 mmol/L. However, a 1.25 mmol/L concentration was administrated for patients with low PTH levels (< 100 pg/mL), and a 1.75 mmol/L concentration was recommended in cases where the PTH levels were high (> 400 pg/mL). Our strategy to optimize the patient’s mineral and bone status during HD has been reported previously [10], and has remained stable for years. The prevention and treatment of SHPT included systematic cholecalciferol supplementation (mean 100 000 IU/month) and individualization of DCC [11] and oral calcium or oral alfacalcidol therapy. Cinacalcet was introduced as a last medical alternative, 7/8 PTX was performed in patients for whom conservative medical therapy failed.

## Statistical analysis

The mean ± standard deviation or median (interquartile range: IQR) were calculated for all variables. We split the patients into two groups according to the development or not of severe SHPT. The baseline characteristics of patients in each group were evaluated using Fisher’s exact test. Logistic regression was applied to evaluate the factors that were associated with severe SHPT. The relative risk for severe SHPT was assessed according to the cut-off value defined by using receiver operating characteristic (ROC) curve analysis for the main modifiable risk factors.

Data were censored for kidney transplantation, death, or transfer to another dialysis centre. Repeated measures analysis of variance was utilized for the serum PTH, calcium and phosphate values at baseline and months 1, 3, 6, 12, 18, 24, 30 and 36. For bone markers (b-ALP and CTX), analysis was performed at baseline and every 6 months.

All statistical analyses were performed using MedCalc software version 11.5.1.0 (MedCalc Software, Ostend, Belgium).

## Results

Between January 2009 and January 2013, 171 patients on HD were evaluated. Initially, 5 patients were excluded due to previous PTX (n = 1) or current antiosteoporotic (n = 1) or cinacalcet therapy (n = 3). Later, 33 patients were excluded because they died or underwent functional kidney grafting within the first 24 months of the study. Finally, 133 patients on HD were included in the analysis and followed up until January 2016. Twenty-eight percent of the patients were women and the mean age was 70.1 ± 14 years old. Of the cohort, 38.5% had diabetes and the mean period of predialysis care under a nephrologist was 32 ± 35 months. From baseline to January 2016, 16.5% (n = 22) of the studied patients received functional kidney grafting, 21% (n = 28) died, and 3.7% (n = 5) moved to another centre.

The dialysis strategy was post-dilutional HDF in 24,6% of cases, and conventional HD in the remaining cases, with a prescribed session time of 3 x 4.6 ± 1 hr (69% underwent dialysis for 4 hr and 18% underwent long dialysis for ≥ 6 hrs.).

**Fig 1:**
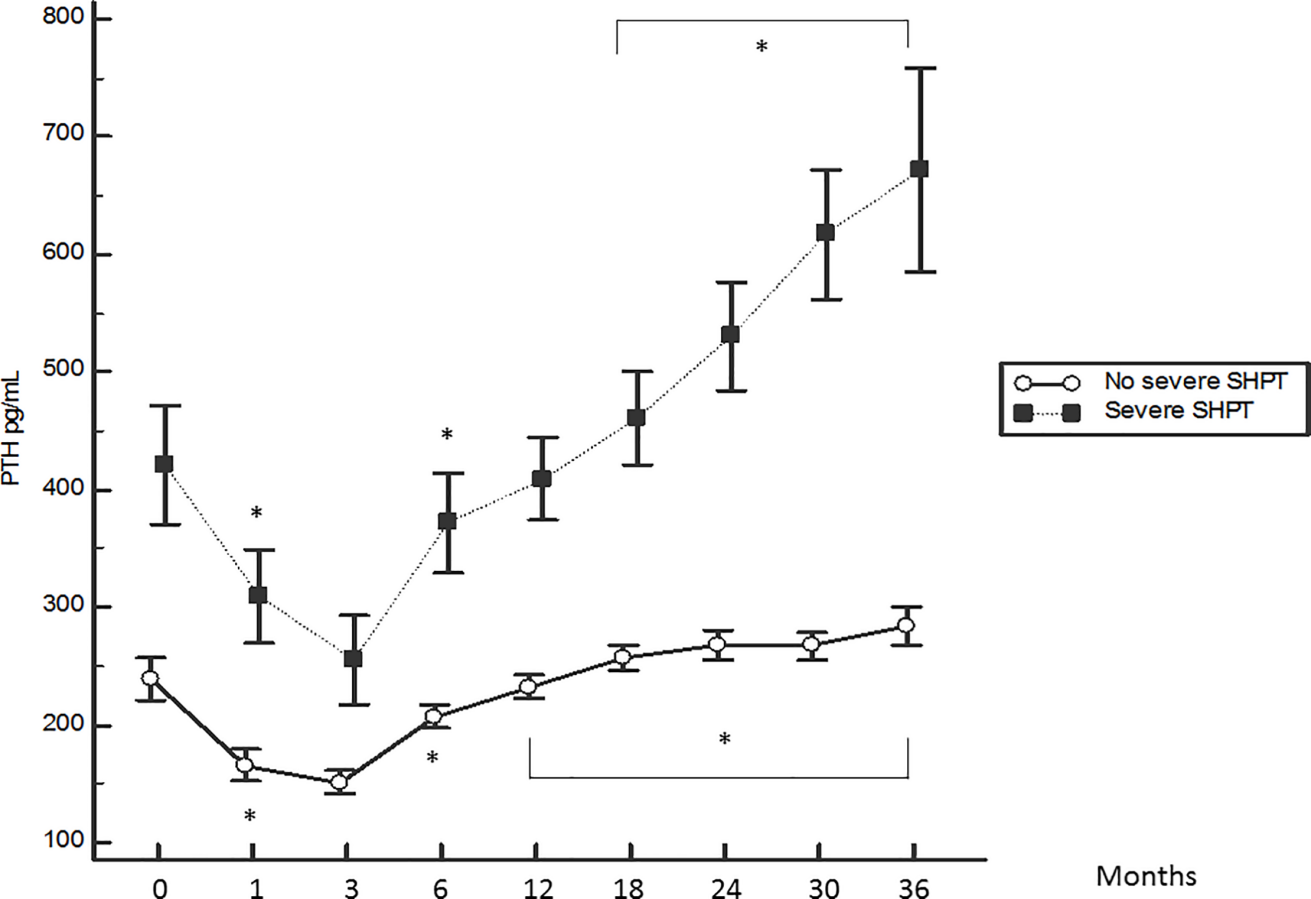
Kinetics of the serum PTH level according to the patient group. *: p< 0.05 with previous month. Mean ± SEM.

**Fig 2:**
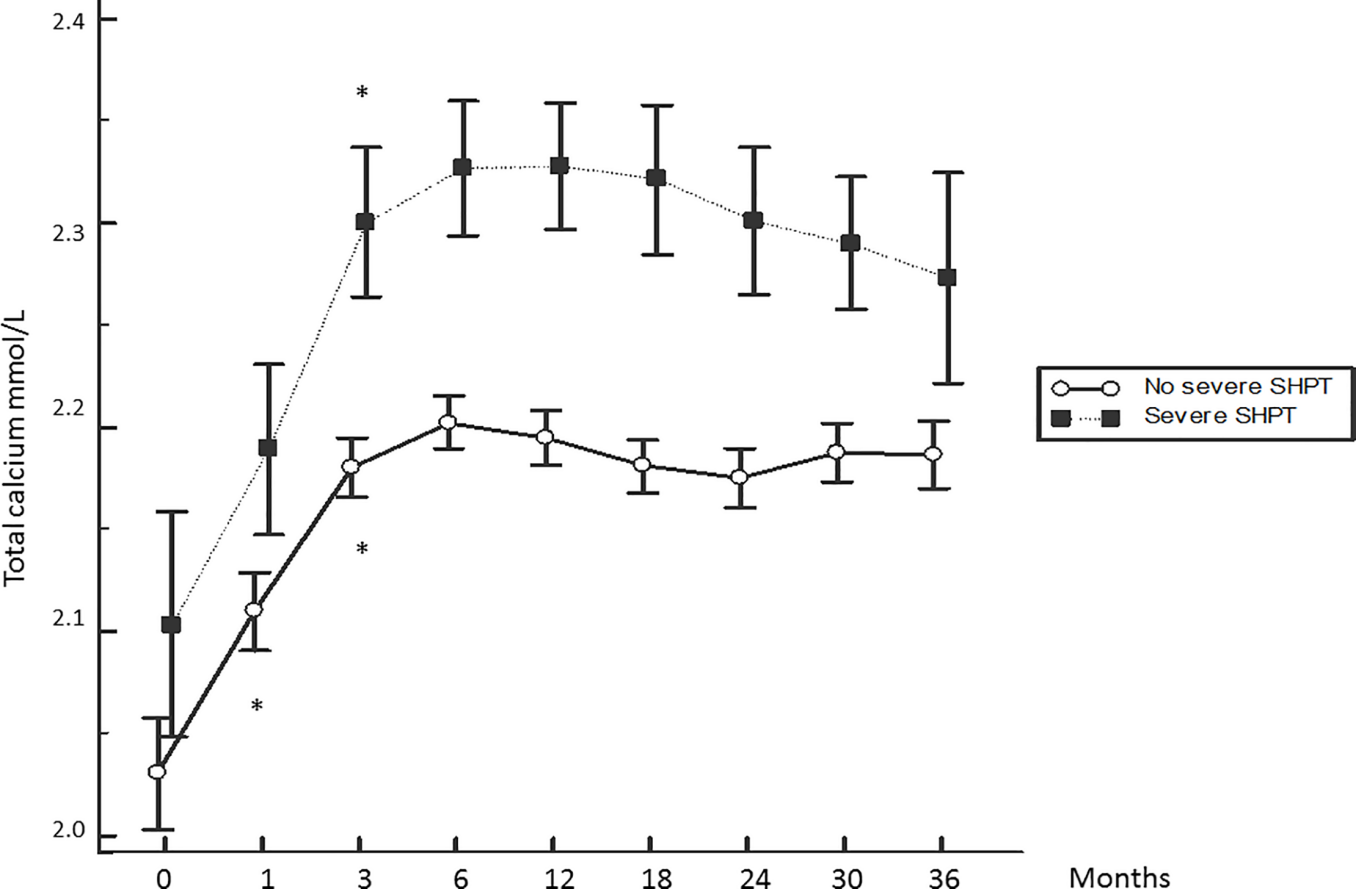
Kinetics of the serum calcium level according to the patient group. *: p< 0.05 with previous month. Mean ± SEM.

**Fig 3:**
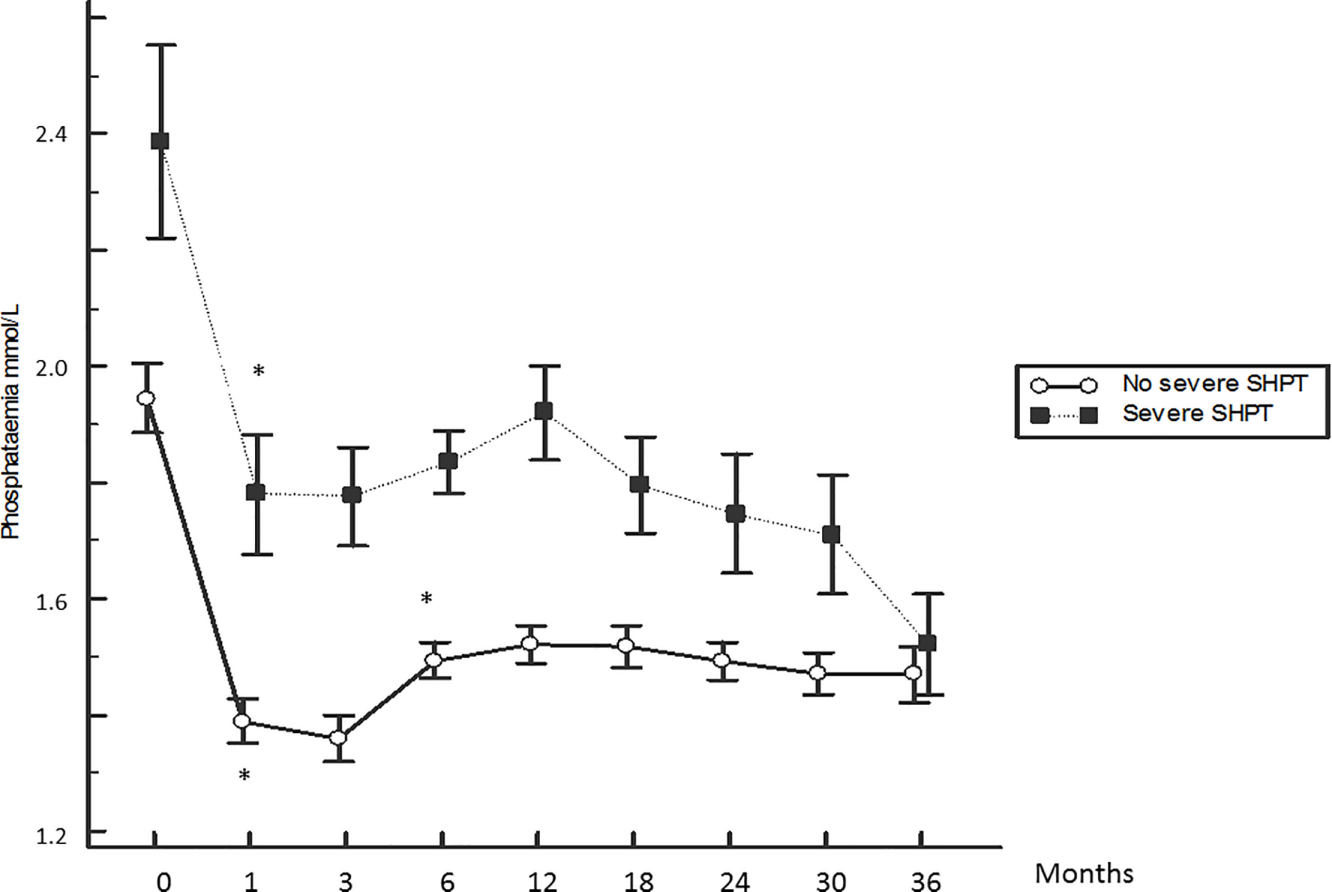
Kinetics of the serum phosphate level according to the patient group. *: p<0.05 with previous month. Mean ± SEM.

The kinetics of the serum PTH, calcium and phosphate levels are displayed in Figs 1, 2 and 3. The phosphate level displayed an initial drop (-25%) between months 1 and 3 and a small increase thereafter, reaching a plateau at approximately 1.5 mmol/L after 12 months before declining again. The serum calcium level displayed a progressive increase until month 6 (+0.9%), reaching a plateau at approximately 2.2 mmol/L thereafter. The mean PTH level at baseline was 275 ± 210 pg/mL, and only 14% of patients had the normal recommended PTH level for CKD stage 5 not on dialysis. PTH displayed an initial drop to the minimum at month 3 (- 41%) and increased progressively until the end of the follow up period (+ 153%).

The kinetics of the serum PTH, calcium and phosphate levels according to the patient group are displayed in Figs 1, 2 and 3. A greater serum PTH level rebound, and higher mean phosphataemia and calcaemia were observed in the group with severe SHPT.

During the study period, severe SHPT was observed in 18.3% of cases: Twenty-four patients were administrated cinacalcet for persistent serum PTH above 800 pg/mL (n = 18), serum PTH > 400 pg/mL with persistent hypercalcaemia (n = 2), or high serum bone marker level (n = 2). Among these patients, 6 required PTX after 6 to 18 months for cinacalcet failure, mainly due to gastro-intestinal side effects at high dosage. In all cases, hyperplasic parathyroid glands were identified. The mean period of time from baseline to cinacalcet prescription was 24 ± 13 months (5 to 50 months). After 30 months, 87% of the initial patients remained free from severe SHPT.

**Fig 4:**
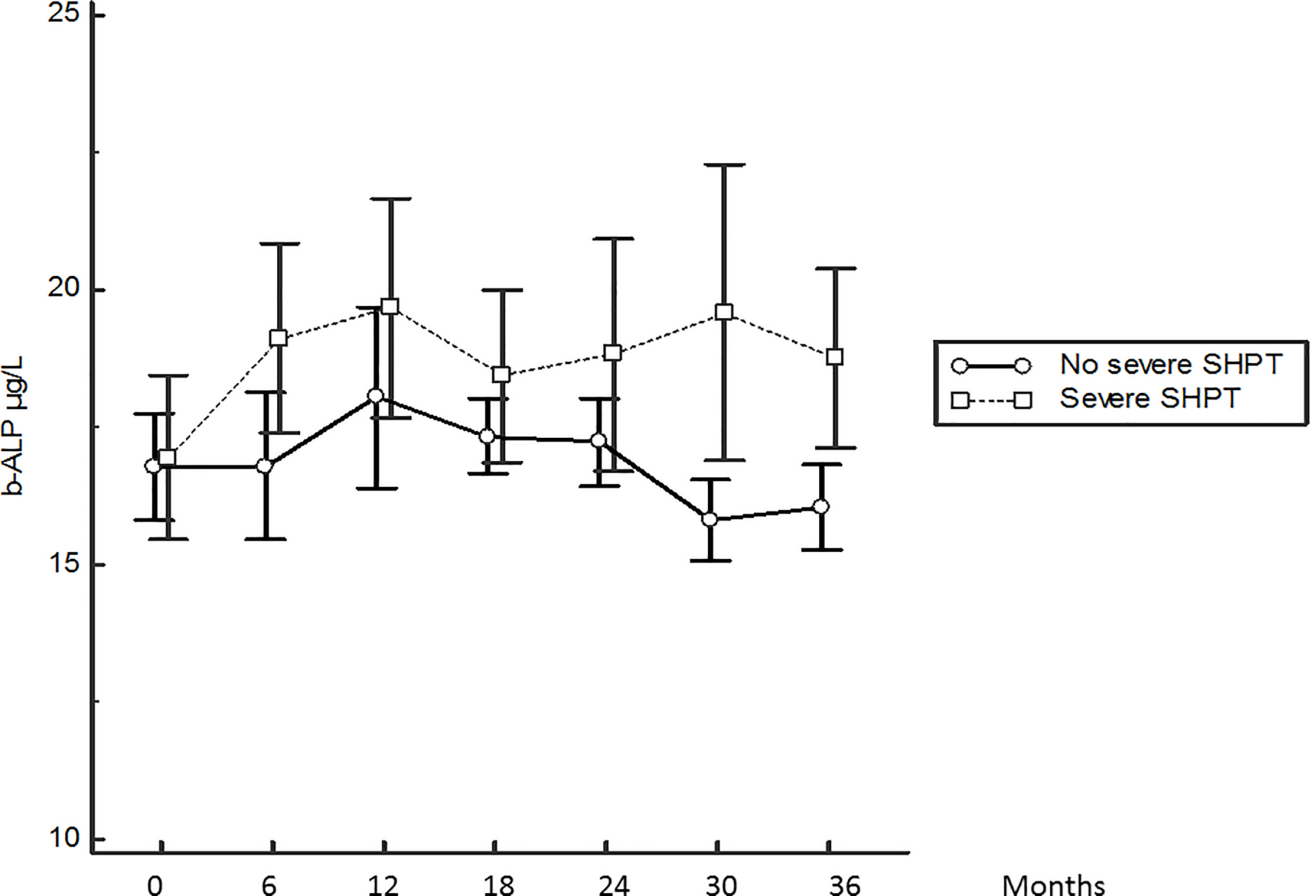
Kinetics of the serum b-ALP level from baseline according to the patient group. Mean ± SEM.

**Fig 5:**
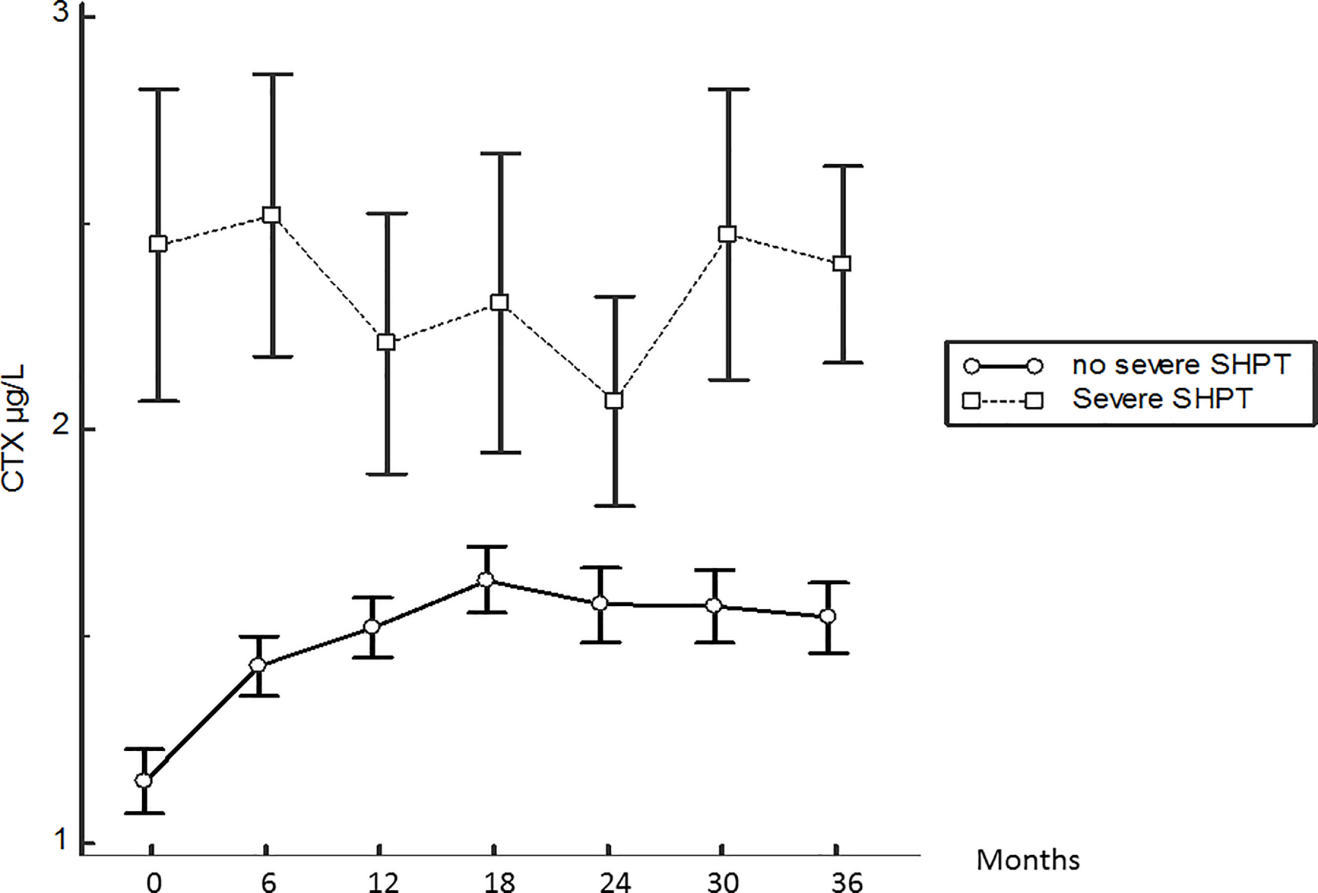
Kinetics of the serum CTX level from baseline according to the patient group. Mean ± SEM. *: p<0.05 = difference with previous value.

Fig 4 displays serum b-ALP changes according to the patient groups. No significant variation from baseline was observed, however, b-ALP became higher in the severe SHPT group only after 24 months. Fig 5 displays serum CTX changes. CTX level was continuously higher in the severe SHPT group. Additionally, the only significant change for CTX was an increase from baseline to month 6 in patients without severe SHPT.

Table 1 reports baseline characteristics of the participants according to the development of future severe SHPT. Patients with the risk of developing severe SHPT were significantly younger (59.6 ± 15 vs. 72.5 ± 13 years), more frequently of female sex (52 vs. 22.7%), less likely to have diabetes (24 vs 41.8%) and cardiac disease (25 vs. 45.9%) and were more often administrated sevelamer (36 vs. 13%) than those who were not at risk for severe SHPT. The only biological differences were higher baseline PTH level, 212 (118–306) pg/ml and 424 (208–550) pg/ml respectively), higher phosphataemia (2.2 ± 0.8 vs. 1.9 ± 0.6 mmol/L), and higher baseline CTX level (2.3 ± 1.9 vs. 1.16 ± 0.8 μg/L, respectively) in those at risk for developing severe SHPT.

**Table 1:** Baseline characteristics of the patients stratified according to a future severe SHPT occurring during the study period. Mean ± SD except for serum CRP and PTH: Median (IQR).

	No severe HPTS N = 109	Severe HPTS N = 24	P
Age (year)	72.5 ± 13	59.6 ± 15	**
Female (%)	22.7	52	**
Nephrologist referral (months)	32 ± 35	30 ± 32	
Diabetes (%)	41.8	24	**
Cardiac disease (%)	45.9	25	**
Peripheral vascular disease (%)	20.3	16	
Cancer (%)	15.6	8	*
Body mass index (kg/m^2^)	25.8 ± 6	25.7 ± 6	
Central venous catheter (%)	39	28	
Creatinine clearance MDRD (ml/min/1.73 m^2^)	8.8 ± 6	5.9 ± 2.6	
Hemoglobin (g/L)	100 ± 13	96 ± 16	
C-reactive Protein (mg/L)	9 (2.7–24)	13 (5–32)	
Albumin (g/L)	32.5 ± 5	31.6 ± 5	
Total calcium (mmol/L)	2.03 ± 0.2	2.1 ± 0.2	
Phosphates (mmol/L)	1.9 ± 0.6	2.2 ± 0.8	*
iPTH (pg/mL)	212 (118–306)	424 (208–550)	*
25-OHD (ng/L)	65 ± 31	58.2 ± 38	
b-ALP (μg/L)	17.1 ± 10	16.9 ± 8	
CTX (μg/L)	1.16 ± 0.8	2.3 ± 1.9	**
Alfacalcidol (%)	20.1	32	
CaCO3 (%)	46	40	
Sevelamer (%)	13	36	*
Cholecalciferol (%)	57.9	60	
ESA (%)	33.9	44	

* p< 0.05

** p< 0.001

In the logistic regression analysis, displayed in Table 2, only female sex, higher baseline serum PTH and CTX levels remained significantly associated with the development of severe SHPT.

**Table 2:** Logistic regression analysis of the baseline factors which were associated with the risk of developing severe SHPT. Age, diabetes, cardiac disease, cancer, phosphataemia and sevelamer use were not significantly associated with the risk for severe SHPT.

Variable	P	OR	CI
Female	0.029	3.2	1.1–10.1
PTH	0.006	1.003	1.0009–1.005
CTX	0.01	1.81	1.12–3.1

Evolution of treatment for SHPT is displayed in Table 3 according to the patient group. Apart from cholecalciferol, which is systematically provided monthly during the first session of each month, calcium salts (mean 1.2 g/day of elemental calcium), alfacalcidol (mean 2.2 μg/week), and dialysate calcium (1.25, 1.5 and 1.75 mmol/L) were adjusted monthly in order to achieve the serum PTH target (150–400 pg/mL). Apart cholecalciferol, both alfacalcidol, calcium salt were more frequently prescribed and dialysate calcium was higher in the group with severe SHPT.

**Table 3:** Evolution of treatments for SHPT according to the patient group.

Months	Baseline	3	12	24	36
**Severe SHPT**					
Cholecalciferol %	60	100	100	100	100
Alfacalcidol %	32 *	32*	32*	68*	71.4*
Calcium salt %	45	36.7*	48*	72*	100*
DCC mmol/L	1.5	1.43 ± 0.1	1.58 ± 0.1	1.62 ± 0.1	1.69 ± 0.1
**No severe SHPT**					
Cholecalciferol %	57.9	100	100	100	100
Alfacalcidol %	21.1	12	15	21	19.7
Calcium salt %	40.3	24	24.7	24.7	27.2
DCC mmol/L	1.5	1.29 ± 0.1*	1.36 ± 0.1*	1.42 ± 0.1*	1.46 ± 0.1*

DCC: Dialysate calcium concentration.

* p< 0.05 = difference between the 2 groups.

The ROC curve analysis of the baseline serum PTH level that was associated with the risk of severe SHPT showed a cut-off value of 374 pg/mL (sensitivity 60%, specificity 85.5%, area under the curve (AUC) 0.72, p< 0.001). For serum CTX, the ROC curve analysis showed a cut-off value of 1.2 μg/L (sensitivity 60%, specificity 80%, AUC 0.76, p< 0.001).

**Fig 6:**
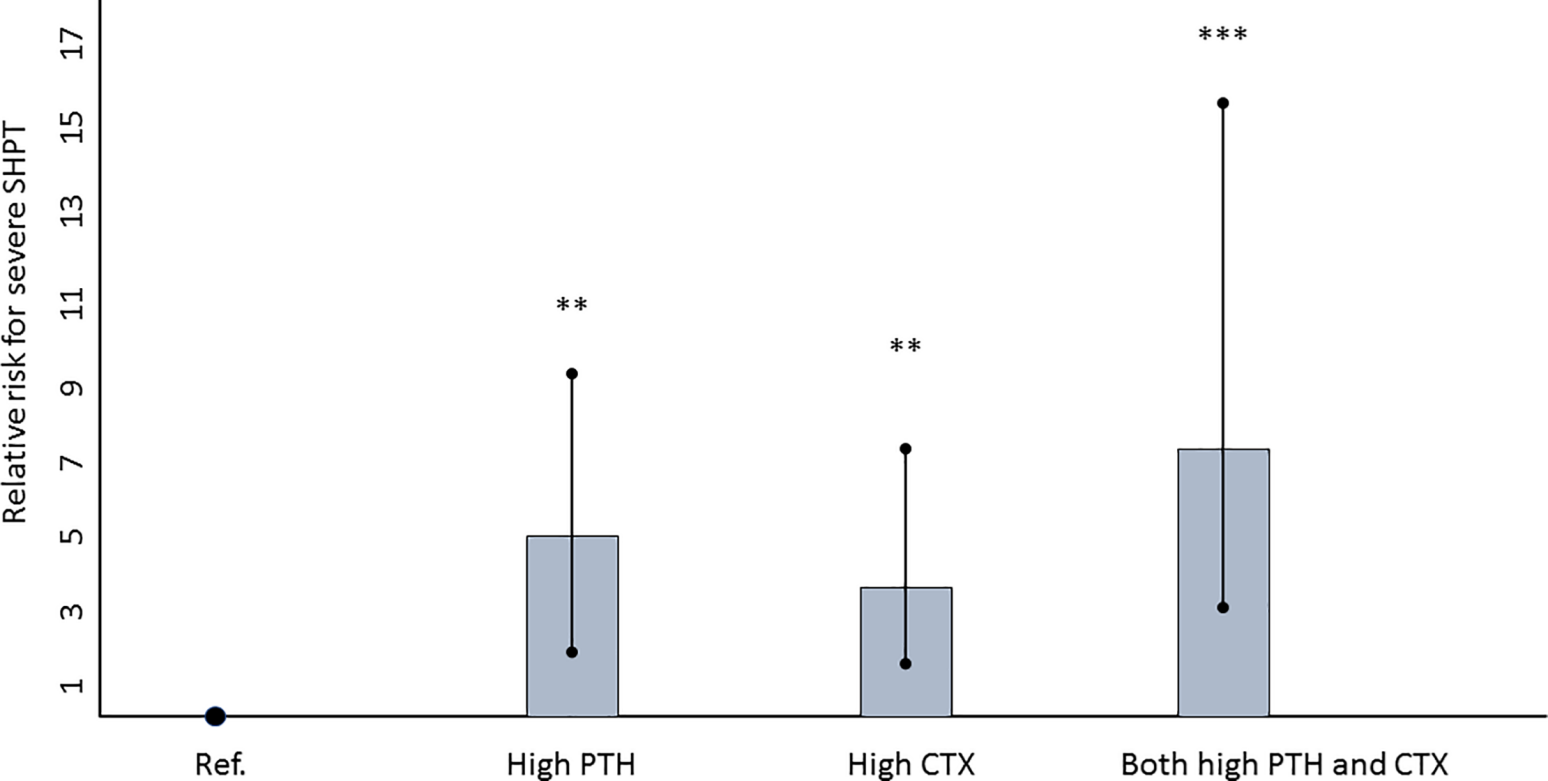
Relative risk of future severe SHPT according to the baseline CTX or / and PTH values. High CTX: > 1.2 μg/L; high PTH: > 374 pg/ml. ** p< 0.001; *** p< 0.0001.

Fig 6 displays the relative risk of future severe SHPT according to the baseline serum CTX and PTH. The relative risk was 3.7 (1.8–7.5, p = 0.002) for baseline CTX > 1.2 μg/L, 4.9 (2.4–9.7, p = 0.001) for baseline PTH > 374 pg/ml, and 7.7 (3.6–16, p< 0.0001) when both criteria were achieved.

All data can be seen in S1 Database and S2 Database.

## Discussion

To our knowledge, this is the first study reporting the evolution of serum PTH, bone markers, calcium and phosphate levels during the first years of HD. In addition, we identified that a high initial serum PTH and CTX values are the main modifiable risk factors associated with the risk for developing severe SHPT in the future.

### Mineral metabolism biological changes

The initial changes in serum PTH, calcium and phosphate levels after the first dialysis session have not been reported. However, changes in serum calcium and phosphate levels is not surprising. Incident patients tend to have hypocalcaemia and this will progressively be improved using a mean DCC of 1.5 mmol/L which allows a positive calcium balance [12].

The evolution of the serum phosphate level is also expected because most patients underwent dialysis with hyperphosphataemia, and dialysis phosphate clearance led to decreased phosphate level. Furthermore, regular blood sampling could lead to optimized phosphate binder prescription.

The change in serum PTH level is more surprising. The initial drop between the first and third months can be explained by the correction of both hypocalcaemia and hyperphosphataemia. The progressive increase of the serum PTH level from month 3 to month 36 is less expected, since individualisation of DCC, alfacalcidol and calcium salts has been applied using the same strategy and is aimed at maintaining a serum PTH level between 150 and 400 pg/mL. Three factors may be involved: a) patients with a low baseline PTH level or having a spontaneous PTH decrease are more likely to die, as we reported recently in the same cohort [13], and we could have selected patients who were exposed to SHPT; b) SHPT began a long time before dialysis and the increasing slope reflects the spontaneous evolution from CKD stage 3 to stage 5 when prevention remains suboptimal; -c) in patients on HD, our therapeutic strategy is insufficient for preventing the increase of serum PTH due to inadequate biological targets or treatment dosage, especially in patients with higher baseline PTH and CTX levels.

### Bone marker evolution

Once again, no data is available concerning the evolution of serum CTX and b-ALP in incident dialysis patients [14]. Specific b-ALP is released by osteoblasts and plays a major role in bone mineralization. B-ALP serum level is independent of kidney function. In our study, overall b-ALP evolution displayed no significant change from baseline in the first 3 years, thus baseline serum b-ALP cannot predict the risk for severe SHPT.

CTX are fragments formed from degradation of type I collagen. They are released during osteoclastic resorption. Serum CTX level is dependent on glomerular filtration rate and dialysis. Apart from the initial increase in CTX value in patients without severe SHPT, which can be due to the progressive increase in serum PTH level from month 3, the CTX evolution displayed no further significant change in the group patients with severe SHPT. Even if bone marker changes are known to be delayed compared to PTH changes, at least after cinacalcet therapy [15], the 3-years evolution should have shown an increase in bone turnover markers associated to the bone consequences of high PTH levels in the severe SHPT group. Additionally, we previously reported that serum CTX and b-ALP evolutions correlated well with one another, but correlated poorly with PTH changes over a 18 months period in prevalent HD patients [16]. Delanaye et al. reported recently that, compared with b-ALP, only CTX correlated well with PTH variation in a short-term period of 6 weeks [17]. We can hypothesize that bone resorption is superior to bone formation in these early stages of SHPT. Thus, CTX seems to be better than b-ALP in predicting severe SHPT despite the most recent recommendation of KDIGO [18], mainly supported by the lack of previous study.

### Risk for severe SHPT

In the present study, we confirmed that SHPT is more frequently observed in female and young patients with a lower incidence of diabetes and cardiovascular disease, as reported in the Dialysis Outcomes Pattern and Practice Study (DOPPS) [19]. Sevelamer is associated with more severe SHPT but this can reflect more severe hyperphosphataemia. However, choosing a non-calcium phosphate binder may lead to the progression of SHPT as observed in a randomized controlled trial of HD [20]. However, in the multivariate analysis of our study, the only modifiable risk factors for severe SHPT were a high baseline serum PTH level (> 374 pg/mL) and high CTX (> 1.2 μg/L) level. It must be highlighted that, because of the huge inter-method variability in PTH measurement, the definition of high baseline PTH value will vary from one PTH assay to another [21]. A high baseline PTH level may reflect the limited quality of predialysis care, including inadequate biological targets and/or the medical strategy that was used. The high baseline CTX level is more questionable since the impact of renal function has been previously reported [22]. It is hypothesized that the renal elimination of CTX is low in CKD stage 5 before dialysis and does not significantly affect its serum level.

The higher phosphataemia level in the group with severe SHPT is not surprising since hyperphosphataemia has been associated with SHPT in most studies. The higher calcaemia level suggest an increase of the calcium-PTH setpoint with some cases of tertiary hyperparathyroidism.

### How to better prevent and treat severe SHPT?

Along with the progression of kidney disease from CKD stage 3 to 5, a progressive decrease in the serum level of vitamin D (25-hydroxy- and 1,25-dihydroxy vitamin D) leads to a negative calcium balance and a positive phosphate balance. However, other factors are also associated with SHPT, including Klotho deficiency, a decrease of fibroblast growth factor (FGF) receptor 1 expression [23] and a decrease in serum 1,25-dihydroxyvitamin D associated with excessive FGF-23 bone synthesis [24]. Indeed, proposed therapies for the treatment of SHPT include native vitamin D[25], oral calcium supplementation [26], increasing the DCC [11], use of calcitriol or its analogues [27], calcimimetics [28] and PTX [29]. SHPT in patients on dialysis is partially associated with the quality of treatment for CKD stages 3–5. In France, Morane et al. reported the results of the *NephroTest* which included approximately 1,000 patients with CKD stages 2 to 5 who were monitored at two centres in Paris [30]. They reported that 59% of patients had SHPT (80% had CKD stages 4 and 5), but only 14% received treatment. Similarly, 8% had hyperphosphatemia, but only 38% of these patients were treated. In a large multicentre study in France of CKD stage 4 and 5, we reported that SHPT, observed in 80% of cases, remained untreated in nearly 50% of cases [31].

Even moderate, SHPT was found to be associated with increased cardiovascular morbidity [32] and mortality [33], and more rapid CKD progression [34]. It therefore seems desirable to comply with the KDIGO recommendations to normalize the serum PTH level before dialysis, even if recent KDIGO recommendations stated that “in patients with CKD G3a–G5 not on dialysis, the optimal PTH level is not known. However, we suggest that patients with levels of intact PTH progressively rising or persistently above the upper normal limit for the assay be evaluated for modifiable factors, including hyperphosphatemia, hypocalcemia, high phosphate intake, and vitamin D deficiency” (2C)[18].

### Limitations

The main limitation of the study is its observational monocentric design, with a relatively small number of patients. As our patients had a homogenous treatment and biological monitoring strategy, these new data should be confirmed in other medical centres and countries.

## Conclusion

In our incident HD cohort, we report the changes in serum calcium and phosphate levels, which tended to normalize during the 3 first months. We also report the changes of the serum PTH level which, after an initial drop, progressively increased toward the initial predialysis value that, when above 5 times the upper limit of the assay, represented the main modifiable risk factor for the development of severe SHPT, together with a high baseline serum CTX level. In contrast, b-ALP was not helpful in identifying severe SHPT. In order to achieve better prevention of SHPT in the earlier CKD stages, use of appropriate therapies should be advocated in order to prevent severe SHPT, which remains difficult to treat during dialysis. In incident HD patients, the association of both high serum PTH and CTX values should lead to maintenance of serum PTH level in the lower range.

## Supporting information

S1 Database (XLS)

S2 Database (XLS)
